# Graphlet-based edge clustering reveals pathogen-interacting proteins

**DOI:** 10.1093/bioinformatics/bts376

**Published:** 2012-09-03

**Authors:** R. W. Solava, R. P. Michaels, T. Milenković

**Affiliations:** Department of Computer Science and Engineering, University of Notre Dame, Notre Dame, IN 46556, USA

## Abstract

**Motivation:** Prediction of protein function from protein interaction networks has received attention in the post-genomic era. A popular strategy has been to cluster the network into functionally coherent groups of proteins and assign the entire cluster with a function based on functions of its annotated members. Traditionally, network research has focused on clustering of nodes. However, clustering of edges may be preferred: nodes belong to multiple functional groups, but clustering of nodes typically cannot capture the group overlap, while clustering of edges can. Clustering of adjacent edges that share many neighbors was proposed recently, outperforming different node clustering methods. However, since some biological processes can have characteristic ‘signatures’ throughout the network, not just locally, it may be of interest to consider edges that are not necessarily adjacent.

**Results:** We design a sensitive measure of the ‘topological similarity’ of edges that can deal with edges that are not necessarily adjacent. We cluster edges that are similar according to our measure in different baker's yeast protein interaction networks, outperforming existing node and edge clustering approaches. We apply our approach to the human network to predict new pathogen-interacting proteins. This is important, since these proteins represent drug target candidates.

**Availability:** Software executables are freely available upon request.

**Contact:** tmilenko@nd.edu

**Supplementary Information:** Supplementary data are available at *Bioinformatics online*.

## 1 INTRODUCTION

Network research spans many domains. We focus on *protein–protein interaction (PPI) networks*, with the goal of identifying new pathogen-interacting proteins. In PPI networks, nodes are proteins and undirected edges correspond to physical binding between the proteins. We focus on PPI networks since it is the proteins, gene products, that carry out most biological processes, and they do so by interacting with other proteins. High-throughput screens for interaction detection, such as yeast two-hybrid (Y2H) assays or affinity purification coupled to mass spectrometry (AP/MS), have yielded partial PPI networks for many model organisms and human (Giot *et al.*, 2003; Simonis *et al.*, 2009; Stelzl *et al.*, 2005; Yu *et al.*, 2008), as well as for bacterial and viral pathogens (LaCount *et al.*, 2005; Parrish *et al.*, 2007). Many biological network datasets are now publicly available (Breitkreutz *et al.*, 2008; Peri *et al.*, 2004).

Analogous to genomic sequence research, biological network research will impact our biological understanding. However, it is in its infancy, owing to computational hardness of many graph theoretic problems (Cook, 1971), as well as to incompleteness of the current network data. Importantly, the number of functionally uncharacterized proteins is large even for well-studied model species (Sharan *et al.*, 2007). Computational characterization of protein function could save resources needed for biological experiments. In particular, PPI network analysis could suggest likely candidates for experimental validation, since proteins aggregate to perform a function, and since PPI networks model these aggregations.

Thus, prediction of protein function (Sharan *et al.*, 2007) and the role of proteins in disease (Goh *et al.*, 2007; Radivojac *et al.*, 2008; Sharan and Ideker, 2008; Vanunu *et al.*, 2010) from PPI networks have received attention in the post-genomic era. It has been argued that proteins that are close in the network are involved in similar biological processes (Sharan *et al.*, 2007) that ‘topologically central’ proteins correspond to ‘biologically central’ (e.g. lethal, aging- or cancer-related) proteins (Jeong *et al.*, 2001; Jonsson and Bates, 2006; Milenković *et al.*, 2011; Sharan and Ideker, 2008) or that proteins with similar topological neighborhoods have similar biological characteristics (Ho *et al.*, 2010; Milenković and Pržulj, 2008).

A particularly popular strategy for functional characterization of proteins has been to *cluster* the network into functionally ‘coherent’ groups of nodes and assign the entire cluster with a function based on functions of its annotated members (Sharan and Ideker, 2008; Sharan *et al.*, 2007). A variety of clustering approaches exist, each with its own (dis)advantages (Brohee and van Helden, 2006; Fortunato, 2010). Typically, they aim to group nodes that are in a dense connected network region (Fortunato, 2010). In addition, approaches exist that cluster ‘topologically similar’ nodes without the nodes necessarily being connected in the network. This is important, since a biological process can have characteristic topological ‘signatures’ throughout the network, not just locally in close network proximity (Ho *et al.*, 2010; Milenković *et al.*, 2010; Milenković and Pržulj, 2008). For example, we designed a measure that computes the topological similarity of the extended network neighborhoods of two nodes, without the nodes necessarily being close in the network (Milenković and Pržulj, 2008). We found that 96% of known cancer gene pairs that are topologically similar according to our measure are actually *not* neighbors in the PPI network; instead, they are at the shortest path distance of up to six (Milenković *et al.*, 2010). As such, they may be missed by approaches that focus on connected nodes only. We clustered proteins in the human PPI network that are topologically similar and showed that function of a protein and its network position are closely related (Milenković and Pržulj, 2008) and that the topology around cancer and non-cancer genes is different (Milenković *et al.*, 2010). We used these observations to predict new cancer genes in melanogenesis-related pathways and our predictions were validated phenotypically (Ho *et al.*, 2010).

**Fig. 1.**
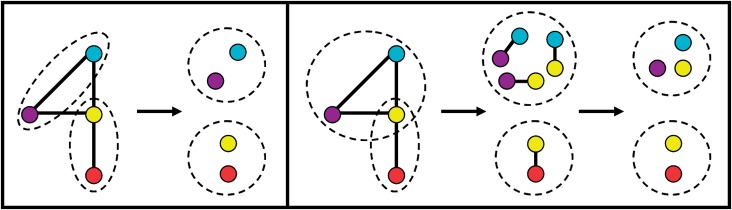
Node clustering (left) versus edge clustering (right)

Traditionally, network research has focused on clustering of *nodes* (Fortunato, 2010). However, a network consists of nodes *and* edges. Hence, why favor nodes over edges, especially when clustering of *edges* may be preferred? Since nodes typically belong to multiple functional groups, and since clusters are expected to correspond to the groups, it may be desirable to allow for a node to belong to multiple clusters. Clustering of nodes typically cannot capture the group overlap, especially if the network is partitioned into disjoint node sets, as is the case with many (although not all) node clustering approaches (Fortunato, 2010). However, clustering of edges can easily capture the group overlap (Fig. 1). Edge clustering was proposed only recently (Ahn *et al.*, 2010; Evans and Lambiotte, 2009). Adjacent (connected) edges that share many neighbors were defined as similar and were thus clustered together (Ahn *et al.*, 2010), outperforming different node clustering methods, including a method that allows for the group overlap. However, it may be of interest to consider edges that are not necessarily adjacent. For example, 97% of pairs of human proteins that both interact with the same pathogen are actually *not* adjacent in the PPI network. In fact, they are at the shortest path distance of up to five. So, they may be missed by approaches that focus on adjacent edges only, since the end nodes of adjacent edges are at most at the distance of two.

Hence, we introduce a new measure of edge similarity that is not only capable of dealing with edges that are not necessarily adjacent, but is also a more sensitive measure of topology than the above shared-neighborhood measure (Ahn *et al.*, 2010). We show that grouping of edges that are similar according to our measure results in clusters of comparable or better quality than the above clustering approach by Ahn *et al.* 2010. We apply our clustering strategy to the human PPI network to identify from the clusters new pathogen-interacting proteins and hence drug target candidates.

### 1.1 Our contribution

**Fig. 2.**
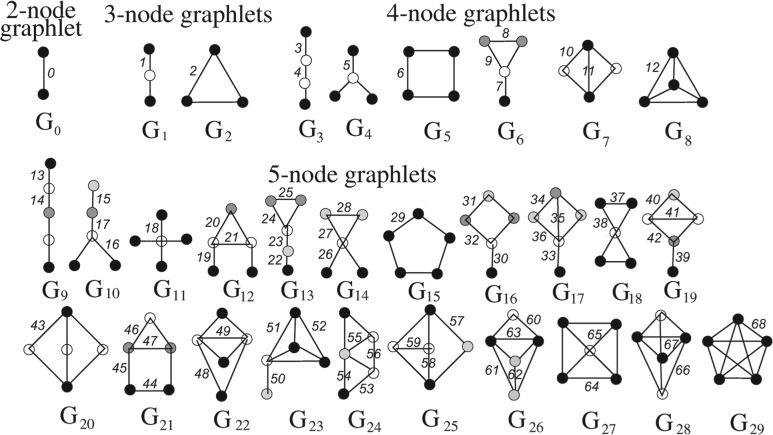
All 2 to 5-node graphlets. They contain 73 topologically unique ‘node orbits.’ In a graphlet, nodes in the same node orbit are of the same shade (Pržulj, 2007). They also contain 69 topologically unique ‘edge orbits.’ (3–5-node graphlets contain 68 edge orbits.) Edge orbits are defined by node orbits of the edges' end nodes (an alternative definition exists; see the main text). In a graphlet, different edge orbits are numbered differently.

We recently designed a graphlet-based measure of the network position of a *node*; graphlets are small *induced* subgraphs (Fig. 2) (Pržulj, 2007). This measure generalizes the degree of a node that counts the number of edges that the node touches (where an edge is the only 2-node graphlet) into the *node graphlet degree vector* (node-GDV) that counts the number of 2 to 5-node graphlets that the node touches (see Section 2). Hence, node-GDV describes the topology of the node's up to 4-deep neighborhood (Milenković and Pržulj, 2008). This is effective: going to distance of four around a node captures a large portion of real networks, as they are small-world (Watts and Strogatz, 1998). For this reason, and since the number of graphlets on *n* nodes increases exponentially with *n*, using larger graphlets could unnecessarily increase the computational complexity. In addition, we designed *node-GDV-similarity* measure to compare node-GDVs of two nodes and quantify the topological similarity of their extended network neighborhoods (Milenković and Pržulj, 2008).

Now, we design *edge-GDV* to count the number of different graphlets that an edge touches (Fig. 2), and we design *edge-GDV-similarity* to compare edge-GDVs of two edges and quantify the topological similarity of their extended network neighborhoods (see Section 2). Unlike the shared-neighborhood measure (Ahn *et al.*, 2010), edge-GDV-similarity can deal with edges independent on whether they are adjacent. In addition, by counting the shared neighbors of end nodes of two (adjacent) edges, the shared-neighborhood measure actually counts the 3-node paths that the end nodes share (Ahn *et al.*, 2010). Since edge-GDV counts the different up to 5-node graphlets that an edge touches, including 3-node paths, edge-GDV is a more constraining measure of topology. See Section 2 for details.

We cluster edge-GDV-similar edges in the human PPI network to predict new *pathogen-interacting (PI) proteins* from the clusters. But first, we compare our approach to existing clustering methods, as follows. The existing edge clustering method mentioned above, henceforth denoted by *edge - shared neighborhood (edge-SN)* (Ahn *et al.*, 2010), was already shown to be superior to different node clustering methods on four baker's yeast PPI networks. With our approach, we cluster edges in the same way as edge-SN, but we use edge-GDV-similarity instead of the edge-SN's shared-neighborhood measure as the distance metric, without changing other aspects of the clustering procedure. This way, we can evaluate the contribution of edge-GDV-similarity alone to the quality of clusters. Just as Ahn *et al.* 2010, we (initially) cluster only adjacent edges, and of all partitions, we choose the one with the maximum density (see Section 2). Just as Ahn *et al.* 2010, we evaluate such partition with respect to: *cluster coverage* (the portion of the network ‘covered’ by ‘non-trivial’ clusters), *overlap coverage* (the amount of node overlap between clusters), *cluster quality* (enrichment of clusters in Gene Ontology (GO) terms (Ashburner *et al.*, 2000)) and *overlap quality* (the correlation between the number of clusters and the number of GO terms that nodes participate in). When applied to the same yeast networks, our approach in comparable or superior to edge-SN (and hence to the node clustering approaches that were outperformed by edge-SN). Thus, we gain by using a more sensitive measure of topology compared to edge-SN. When we cluster both adjacent *and* non-adjacent edges, our method in general performs even better. Hence, we gain further by using a measure that can deal with edges that are not necessarily adjacent. We note that we do not propose a new clustering method but a new edge similarity measure that can serve as a distance metric for existing clustering methods.

After we evaluate our approach on yeast, we apply it to human, hypothesizing that if many end nodes of edges in a cluster are PI proteins, then the other end nodes of edges in that cluster are likely PI proteins. Here, instead of comparing different distance metrics within the same clustering method (as above), we aim to compare different clustering methods when using the same (best) distance metric. Hence, we use edge-GDV-similarity as the distance metric for two popular clustering methods: hierarchical and *k*-means (i.e. *k*-medoids) clustering (see Section 2). In addition, we aim to evaluate how much we gain by clustering edges compared to clustering nodes. This is partially answered on yeast, since we compare our method to edge-SN, which was already compared to different node clustering methods on the same networks. However, our edge clustering method (and hence edge-SN) is conceptually different than these node clustering methods. For a fair comparison of edge and node clustering, we use edge-GDV-similarity and node-GDV-similarity, conceptually *equivalent* measures of the network position of an edge and a node, respectively, as distance metrics for the same clustering method. We do this for both hierarchical and *k*-medoids clustering.

Hence, we apply four clustering strategies to human to predict new PI proteins: hierarchical node clustering (node-HIE), hierarchical edge clustering (edge-HIE), *k*-medoids node clustering (node-KM) and *k*-medoids edge clustering (edge-KM). We evaluate their prediction accuracies in systematic leave-one-out cross-validation and precision–recall settings (see Section 2). Interestingly, edge-HIE is superior, followed by node-KM, edge-KM and node-HIE. Since all clustering strategies except node-HIE produce non-random results, for each of them, we identify clusters that are statistically significantly enriched in known PI proteins and predict novel PI proteins from the clusters. This way, we complement predictions produced by the different strategies. We study their overlap and identify ‘high-confidence’ predictions produced by multiple strategies. We validate 44% of our predictions in the literature.

## 2 METHODS

### 2.1 Datasets

**Yeast PPI networks.** We cluster the same four baker's yeast PPI networks that edge-SN was evaluated on (Ahn *et al.*, 2010; Yu *et al.*, 2008): i) *Y2H* network, obtained by Y2H, with 1647 proteins and 2518 PPIs; ii) *AP/MS* network, obtained by AP/MS, with 1004 proteins and 8319 PPIs; iii) *LC* network, obtained by literature curation, with 1213 proteins and 2556 PPIs and iv) *ALL* network, representing the union of Y2H, AP/MS and LC, with 2729 proteins and 12 174 PPIs. Using these different networks ensures that our method is robust to different types of experiments for PPI detection.

**The human PPI network.** The human PPIs were obtained in July 2011 from BioGRID (version 3.1.79) (Breitkreutz *et al.*, 2008) and HPRD (Peri *et al.*, 2004). We take the union of the two networks (using UniProt protein IDs (The UniProt Consortium, 2012)) to increase the coverage of the cellular space, resulting in 12 111 unique proteins and 59 191 unique PPIs.

**Pathogen-interacting proteins.** 9884 human–pathogen PPIs were obtained from VirusMint (Chatr-aryamontri *et al.*, 2009) and Dyer *et al.*, 2008. The PPIs involve 1338 human proteins, of which 1113 are present in the human PPI network, and 706 pathogen proteins from 193 pathogens.

### 2.2 Related work

We compare our method to three popular node clustering methods: clique percolation (Palla *et al.*, 2005), greedy modularity optimization (Newman, 2004) and Infomap (Rosvall and Bergstrom, 2008). In addition, we compare it to the existing edge clustering algorithm, edge-SN (Ahn *et al.*, 2010). Briefly, *clique percolation* is the most prominent overlapping node clustering algorithm, *greedy modularity optimization* is the most popular modularity-based technique, and *Infomap* is often considered the most accurate method available (Ahn *et al.*, 2010). *Edge-SN* hierarchically groups adjacent edges whose non-common end nodes share many neighbors (see below). We did not run these algorithms on the yeast networks ourselves. Instead, we use the results reported by Ahn *et al.* (2010) who ran the algorithms on the same networks. For details on how the methods were implemented, see Ahn *et al.* (2010). We do explain how Ahn *et al.* (2010) implemented edge-SN, as we implement our method in the same way (except that we use a different distance metric).

Edge-SN algorithm works as follows. If the set of node *i* and its neighbors is denoted as *n*(*i*), the similarity between adjacent edges *e*_*ik*_ and *e*_*jk*_ with common node *k* is *S*(*e*_*ik*_ ,*e*_*jk*_) = |*n*(*i*)∩*n*(*j*)|*/*|*n*(*i*)∪*n*(*j*)|. This *shared-neighborhood measure* is used as a distance metric for single-linkage *hierarchical clustering*. With this method, a tree or dendrogram, is created. Leaves of the tree are edges of the network and an interior node in the tree represents a cluster made up of all children of the node. The tree is constructed by assigning each edge to its own cluster and iteratively merging the most similar pair of clusters. The tree has to be cut in order to create a partition of *K* clusters. To determine where to cut the tree, edge-SN uses an objective function called *partition density*, computed as follows. For a network with *M* edges, {*P*_1_,··· ,* P*_*K*_ } is a partition of the edges into *K* clusters. Cluster *C* has *m*_*C*_ = |*C*| edges and *n*_*C*_ = |∪ _*e*_*ij*_∈*C*_ *i,j*| nodes. *C*'s density is *D*_*C*_ = [*m*_*C*_ – (*n*_*C*_ – 1)]*/*[*n*_*C*_ (*n*_*C*_ – 1)*/*2–(*n*_*C*_ – 1)] and the partition density is *D*= (2*/M*) Σ_*K*_ [*m*_*C*_* –*(*m*_*C*_ – (*n*_*C*_ – 1))*/*((*n*_*C*_ – 2)(*n*_*C*_ – 1))]. For details, see Ahn *et al.* (2010). Edge-SN cuts the tree at different levels and chooses a partition with the maximum value of *D*. However, meaningful structure may also exist above and below the level corresponding to maximum *D* (Ahn *et al.*, 2010).

### 2.3 New measures of network topology: edge graphlet degree vector (edge-GDV) and edge-GDV-similarity

A graphlet is an *induced* subgraph of graph *X* that contains *all* edges of *X* connecting its nodes (Fig. 2). We generalized the degree of node *v* that counts the number of edges that *v* touches (where an edge is the only 2-node graphlet, *G*_0_ in Fig. 2) into *node graphlet degree vector* (node-GDV) of *v* that counts the number of 2–5-node graphlets (*G*_0_, *G*_1_, ..., *G*_29_ in Fig. 2) that *v* touches (Milenković and Pržulj, 2008). We need to distinguish between *v* touching, e.g. a *G*_1_ at an end node or at the middle node, since *G*_1_ admits an automorphism that maps its end nodes to each other and the middle node to itself. To understand this, recall the following. An isomorphism *f* from graph *X* to graph *Y* is a bijection of nodes of *X* to nodes of *Y* such that *xy* is an edge of *X* if and only if *f* (*x*)*f* (*y*) is an edge of *Y*. An automorphism is an isomorphism from *X* to itself. The automorphisms of *X* form the automorphism group, Aut(*X*). If *x* is a node of *X*, then the automorphism node orbit of *x* is Orb(*x*)= {*y* ∈ *V* (*X*)|*y* = *f* (*x*) for some *f* ∈ Aut(*X*)}, where *V* (*X*) is the set of nodes of *X*. Thus, end nodes of a *G*_1_ belong to one node orbit, while its middle node belongs to another one. There are 73 node orbits for 2–5-node graphlets. Hence, node-GDV of *v* has 73 elements counting how many node orbits of each type touch *v* (*v*'s degree is the first element). It captures *v*'s up to 4-deep neighborhood and thus a large portion of real networks, as they are small-world (Watts and Strogatz, 1998).

Since a graphlet contains nodes *and* edges, we propose a new graphlet-based measure of the network position of an *edge*. We define *edge-GDV* to count the number of graphlets that an edge touches at a given ‘edge orbit’ (Fig. 2). We define edge orbits as follows. Given the automorphism group of graph *X*, Aut(*X*), if *xy* is an edge of *X*, then the edge orbit of *xy* is Orb_*e*_(*xy*) = {*zw* ∈ *E*(*X*)|*z* = *f* (*x*) and *w* = *f* (*y*) for some *f* ∈ Aut(*X*)}, where *E*(*X*) is the set of edges of *X*. Alternatively, we can define edge orbits as follows. An edge-automorphism from graph *X* to graph *Y* is a bijection *g* of edges of *X* to edges of *Y* such that two edges *xy* and *zw* share a node in *X* if and only if edges *g*(*xy*) and *g*(*zw*) share a node in *Y*. An edge-automorphism is an edge-isomorphism from *X* to itself. The edge-automorphisms of *X* form the edge-automorphism group, *Aut*_*e*_(*X*). If *xy* is an edge of *X*, then the edge orbit of *xy* is Orb_*e*_(*xy*) = {*zw* ∈ *E*(*X*)|*zw* = *g*(*xy*) for some *g* ∈ *Aut*_*e*_(*X*)}. Independent of which of the two definitions we choose, the resulting edge orbits are the same. For example, in Fig. 2, in a *G*_1_, both edges are in edge orbit 1. In a *G*_2_, all three edges are in edge orbit 2. In a *G*_3_, the two ‘outer’ edges are in edge orbit 3, while the ‘middle’ edge is in edge orbit 4, and so on. There are 68 edge orbits for 3 to 5-node graphlets. (We intentionally exclude edge orbit 0 in the only 2-node graphlet, *G*_0_, as each edge touches exactly one *G*_0_, i.e. itself.)

Comparing edge-GDVs of two edges gives a sensitive measure of their topological similarity, since their *extended* network neighborhoods are compared. Using some existing measure, e.g. Euclidean distance, to compare edge-GDVs might be inappropriate, as some edge orbits are not independent. Instead, we design edge-GDV-similarity measure as follows. For an edge *e*, *e*_*i*_ is the *i*^th^ element of its edge-GDV. The distance between the *i*^th^ edge orbits of edges *e* and *f* is *D*_*i*_ (*e, f*)= *w*_*i*_ ×|*log*(*e*_*i*_ + 1)–*log*(*f*_*i*_ + 1)|*/log*(*max*{*e*_*i*_,*f*_*i*_}+ 2), where *w*_*i*_ is the weight of edge orbit *i* that accounts for edge orbit dependencies. For example, the differences in counts of orbit 2 of two edges will imply the differences in counts of all other orbits that contain orbit 2, such as orbits 8–12 (Fig. 2). This is applied to all edge orbits: the smaller the number of orbits that affect orbit *i* (including itself), *o*_*i*_, the higher its weight *w*_*i*_, where *w*_*i*_ = 1–*log*(*o*_*i*_)*/log*(68). Clearly, *w*_*i*_ is in (0, 1] and the highest weight of 1 is assigned to orbit *i* with *o*_*i*_ = 1. The *log* is used in the formula for *D*_*i*_ because the *i*^th^ elements of two edge-GDVs can differ by several orders of magnitude and we do not want the distance between edge-GDVs to be dominated by large values; in addition, we want to account for the relative difference between *e*_*i*_ and *f*_*i*_ and that is why we divide by the value of the denominator, which also scales *D*_*i*_ to [0, 1). The constants are added to prevent *D*_*i*_ to be infinite. The total distance is 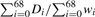. Finally, edge-GDV-similarity is *S*(*e, f*)= 1–*D*(*e, f*). It is in (0, 1]. The higher the edge-GDV-similarity, the higher the topological similarity of edges' extended network neighborhoods. We design edge-GDV-similarity as described because we already designed node-GDV-similarity, which compares node-GDVs, in a similar way (Milenković and Pržulj, 2008), and because we showed in different contexts that node-GDV-similarity successfully extracts function from network topology (Kuchaiev *et al.*, 2010; Memisević *et al.*, 2010; Milenković *et al.*, 2010, 2011). So, we expect edge-GDV-similarity to successfully extract function from topology as well.

### 2.4 Our clustering strategies

**Clustering of the yeast PPI networks.** We cluster the yeast PPI networks in the same manner as edge-SN, except that we use edge-GDV-similarity as the distance metrics instead of using the shared-neighborhood measure. Initially, for a fair comparison with edge-SN, we cluster adjacent edges only, to test if and how much we gain by using our more sensitive measure of edge similarity. Later on, we cluster all edges, to test if and how much we gain by taking into account edges that are not necessarily adjacent. Some further information is provided below, after defining measures of partition quality.

**Clustering of the human PPI network.** We cluster the human PPI network to identify novel PI proteins from the clusters. To test if and how much we gain by clustering edges instead of nodes, we use edge-GDV-similarity as the distance metric to cluster edges, and we use node-GDV-similarity as the distance metric to cluster nodes. We do this for two popular clustering methods: hierarchical clustering and *k*-medoids clustering. With all four clustering strategies (hierarchical node clustering, hierarchical edge clustering, *k*-medoids node clustering and *k*-medoids edge clustering), we cluster nodes/edges independent on whether they are adjacent. When node-/edge-GDV-similarities are computed, all proteins and PPIs in the human network are considered. However, in the clustering process and in subsequent analyses of the clusters, we consider only nodes with more than three interacting partners in the network; consequently, we consider only edges with both end nodes having degree of more than three. We do this since poorly connected proteins are more likely to be involved in noisy PPIs. Similar was done previously (Brun *et al.*, 2004; Ho *et al.*, 2010; Milenković *et al.*, 2010). In the network, there are 6121 proteins with degrees higher than three (of which 948 are PI proteins) and there are 47 735 edges between these proteins.

We form hierarchical tree as described above, clustering both adjacent and non-adjacent nodes/edges. We cluster non-adjacent nodes/edges because we hypothesize that function is encoded throughout the network, not just in dense connected local network regions. For this reason, choosing a partition with the maximum partition density might be inappropriate. Instead, we choose a partition as follows. We already clustered the human PPI network into hierarchical node clusters and tested many values for the desired number of clusters, *K*_HIE_: 100, 250, 500, 750, 1000, 1250, 1500, 1750, 2000, 2250 and 2500 (Milenković *et al.*, 2010). *K*_HIE_ of 1250 resulted in the best overall precision-recall (see Milenković *et al.* (2010) for details). Hence, we use *K*_HIE_ = 1250 in this study for hierarchical node clustering. For a fair comparison of node and edge clustering, we use the same *K*_HIE_ for hierarchical edge clustering. Using the same *K*_HIE_ for node and edge clustering will tell us if we gain by allowing for the group overlap with edge clustering.

We form *k*-medoids (KM) node/edge clusters as follows. KM is a modification of the *k*-means algorithm that chooses *actual data points* as centers. We choose the value for the desired number of *k*-medoids clusters, *K*_KM_, as follows. We already clustered the human PPI network into *k*-medoids node clusters and tested many values for *K*_KM_: 100, 250, 500, 750, 1000, 1250, 1500, 1750, 2000, 2250 and 2500 (Milenković *et al.*, 2010). The algorithm could not converge for *K*_KM_ of 1500 or higher, and it produced inconsistent clusters over multiple runs for *K*_KM_ of 750 or lower. Of the remaining two, *K*_KM_ of 1000 resulted in the best overall precision–recall (see (Milenković *et al.*, 2010) for details). Hence, we use *K*_KM_ = 1000 in this study for *k*-medoids node clustering. For a fair comparison of node and edge clustering, we use the same *K*_KM_ for *k*-medoids edge clustering.

### 2.5 Quality of partitions

**Yeast.** We evaluate a partition with respect to the same measures that were used by edge-SN: cluster coverage (CC), overlap coverage (OC), cluster quality (CQ) and overlap quality (OQ). CC is the fraction of nodes that belong to at least one ‘non-trivial’ cluster of three or more nodes. OC is the average number of non-trivial clusters that nodes belong to. CQ is the ratio of the average GO (Ashburner *et al.*, 2000) similarity over all node pairs that are in the same cluster and the average GO similarity over all node pairs in the network. OQ is the mutual information between the number of GO terms and the number of non-trivial clusters that proteins belong to. Raw values for the four measures do not necessarily fall in [0, 1]. Hence, just as Ahn *et al.*, 2010, we normalize each measure such that the best method has a value of one. Then, the overall partition quality is the sum of these four normalized measures, such that the maximum achievable score is four.

We can now note the following. To mimic (Ahn *et al.*, 2010), we would report the partition with maximum partition density *D*. However, we find that CC is strongly negatively correlated with CQ and OQ, and sometimes with OC, over all of our partitions (Supplementary Fig. S1). Thus, choosing the partition with low CC would result in high CQ and OQ (and sometimes OC), hence artificially increasing the overall partition quality. Since in three out of four yeast networks CC is lower for edge-SN than for the node clustering methods, it might not be surprising that edge-SN's overall partition quality is the highest. Analogously, since edge-SN's partitions with maximum *D* have lower CC than our partitions with maximum *D*, our partitions may have lower overall partition quality simply because of the strong negative correlation between CC and other measures. Hence, we find the partition with maximum *D* among all partitions that have CC less than or equal to CC of edge-SN's partition with maximum *D*. Then, we report either the partition obtained in this way or the partition with maximum *D* (independent of its CC), whichever has better overall partition quality. When we cluster both adjacent and non-adjacent edges, selecting the partition based on its density, as just described, might be inappropriate (see above). Thus, in this case, we also report the partition with the best overall partition quality.

**Human.** For each cluster, for each pathogen, we measure the enrichment of the cluster in proteins that interact with the pathogen. If the enrichment is statistically significant (see below), for each protein in the cluster, we use *leave-one-out cross-validation* (Sharan *et al.*, 2007) by ‘hiding’ whether the protein is known to interact with the pathogen and predicting it as interacting with the pathogen if the enrichment of the cluster is above a given threshold *k*. We vary *k* from 0% to 100%, in increments of 1%. For each *k*, we evaluate the prediction accuracy through precision (a measure of exactness) and recall (a measure of completeness), combined into *F*-score. Given our predictions, *precision* is the number of true positives out of both true positives and false positives, *recall* is the number of true positives out of both true positives and false negatives, and *F-score* = 2·Precision·Recall*/*Precision+Recall. We compute *F*-scores for all four clustering methods over the entire range for *k*. As a combination of precision and recall, *F*-score makes method comparison easier.

**Statistical significance.** When we compute enrichments, we consider as PI proteins all human proteins (with degrees above three; see above) that interact with a pathogen that interacts with at least one other human protein (with degree above three). (Hence, we consider only pathogens that interact with at least two human proteins. If we considered a pathogen that interacts with only one protein, we could never predict other proteins to interact with that pathogen, since we can measure enrichment only when at least two proteins in the cluster interact with the pathogen; but this one protein would be lowering recall as a false positive.) There are 936 such PI proteins.

We compute *p*-value of a given enrichment as follows. There are |*N*| PI proteins (as just defined) in the network; |*P*| out of |*N*| PI proteins interact with a given pathogen; |*C*| out of |*N*| PI proteins are in a given cluster; |*p*| out of |*C*| proteins in the cluster interact with the given pathogen. The enrichment is |*p*|*/*|*C*|. The *p*-value is obtained by the hypergeometric model for sampling without replacement: 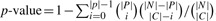. An enrichment with *p*-value below 0.05 is statistically significant.

We assess the statistical significance of observing *F*-scores computed from data clusters by comparing them with *F*-scores computed from random clusters. We create random clusters by randomly assigning nodes to clusters of the same size as the data clusters. We compute *F*-scores on such randomized clusters as described above, averaged over 100 randomization runs.

## 3 RESULTS AND DISCUSSION

We cluster edge-GDV-similar edges in PPI networks. We compare our approach with other methods (Section 3.1). Then, we use it to identify new pathogen-interacting (PI) proteins (Section 3.2).

### 3.1 Comparison with other methods on yeast

**Fig. 3.**
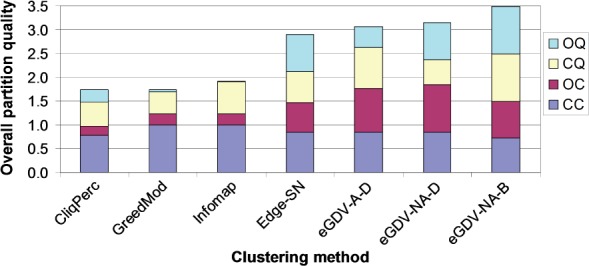
Method comparison for AP/MS yeast PPI network

We evaluate three existing node clustering methods and one existing edge clustering method against three versions of our method (Table 1) on four yeast PPI networks (Y2H, AP/MS, LC and ALL), with respect to four partition quality measures (cluster coverage – CC, overlap coverage – OC, cluster quality – CQ and overlap quality –OQ) that are combined into the normalized overall partition quality; see Section 2. Results are shown in Fig. 3 for AP/MS network and in Supplementary Fig. S2 for Y2H, LC and ALL networks.

**Table 1.** Different clustering approaches evaluated in this study on the yeast PPI networks

Method	Description
CliqPerc	Clique percolation (Palla *et al.*, 2005)
GreedMod	Greedy modularity optimization (Newman, 2004)
Infomap	Infomap (Rosvall and Bergstrom, 2008)
Edge-SN	Edge - shared neighborhood (Ahn *et al.*, 2010)
eGDV-A-D	Our method when clustering **a**djacent edges only and reporting the partition with the maximum **d**ensity
eGDV-NA-D	Our method when clustering adjacent and **n**on-**a**djacent edges and reporting the partition with the maximum **d**ensity
eGDV-NA-B	Our method when clustering adjacent and **n**on-**a**djacent edges and reporting the partition with the **b**est overall quality

CliqPerc, GreedMod and Infomap are existing node clustering approaches. Edge-SN is an existing edge clustering approach. See Section 2 for details.

We gain by using edge-GDV-similarity for clustering: eGDV-A-D outperforms all node clustering approaches on all networks. (This includes node clustering by using node-GDV-similarity, as shown in Supplementary Fig. S3.) Also, it outperforms edge-SN on Y2H and AP/MS. Although edge-SN is slightly better than and comparable to eGDV-A-D on LC and ALL networks, respectively, eGDV-NA-D outperforms edge-SN on these two networks, as well as on AP/MS. Hence, we gain further by clustering non-adjacent edges in addition to adjacent ones. The only exception is Y2H, for which edge-SN is slightly better than eGDV-NA-D. However, as already noted, eGDV-A-D outperforms edge-SN on Y2H network. Hence, we are always superior, with either eGDV-A-D or eGDV-NA-D or both eGDV-A-D and eGDV-NA-D. With eGDV-NA-B, we further demonstrate our superiority over all other methods on all networks.

### 3.2 Prediction of pathogen-interacting human proteins

**Motivation.** Of all pairs of PI human proteins that both interact with the same pathogen, 96.8% are *not* adjacent in the network: 3.2%, 25.8%, 55.8%, 14.8% and 0.4% of them are at the shortest path distance of 1, 2, 3, 4 and 5, respectively. In addition, topologies around PI proteins that interact with the same pathogen are different than topologies around PI proteins that interact with different pathogens, as well as than topologies around non-PI proteins. That is, PI proteins that interact with the same pathogen are more node-GDV-similar to each other than to PI proteins interacting with different pathogens or to non-PI proteins (Supplementary Section S1). Thus, we cluster topologically similar but not necessarily adjacent network regions to predict new PI proteins from clusters enriched in known PI proteins. To study how the choice of the clustering method affects the predictions, we cluster both edge-GDV-similar edges and node-GDV-similar nodes, with both hierarchical and *k*-medoids clustering. We denote hierarchical node clustering as node-HIE, *k*-medoids node clustering as node-KM, hierarchical edge clustering as edge-HIE and *k*-medoids edge clustering as edge-KM.

**Cluster properties.** Cluster sizes, numbers of connected components, and average shortest path distances are shown in Supplementary Fig. S4. With node-HIE, cluster sizes follow a ‘power-law’: many nodes are in small clusters (e.g. 1144 (18.7%) of the nodes are in trivial clusters of size 1 or 2), but there exist some large clusters (e.g. there are four clusters with more than 200 nodes). On the other hand, with node-KM, only 121 (2%) of the nodes are in trivial clusters, the majority of clusters have size 3–20, only two have size 21–50, and there are no larger clusters. With edge-HIE and edge-KM, almost no nodes are in trivial clusters. Non-trivial cluster sizes follow a ‘power-law’ for edge-HIE. For edge-KM, they follow a ‘normal-like’ distribution, with the majority of clusters having size 26–150. Since edge-KM results in more larger clusters than edge-HIE, it is not surprising that the average node membership in clusters is twice larger for edge-KM (Supplementary Fig. S5).

With node-HIE, even the smallest non-trivial clusters consist of multiple connected components. As the cluster size increases, so does the number of connected components in the cluster. The same is observed for node-KM. With edge-HIE, many of the smallest non-trivial clusters consist of a single connected component, but then again the number of connected components in the cluster increases with the increase in cluster size. Surprisingly, there are some edge-HIE clusters of size more than 200 that consist of a single component. Even more surprisingly, with edge-KM, larger clusters (26–200 nodes) tend to consist of single connected components more often than small clusters (6–25 nodes). However, there exist larger edge-KM clusters that consist of multiple components. Importantly, multi-component clusters would be missed by approaches that can deal with adjacent edges only (e.g. edge-SN). 1-component edge-HIE clusters tend to have smaller average shortest path distances than 1-component edge-KM clusters. This is not surprising, since 1-component edge-HIE clusters are mostly small (3–5 nodes), whereas 1-component edge-KM clusters are mostly large (26–150 nodes).

**Fig. 4.**
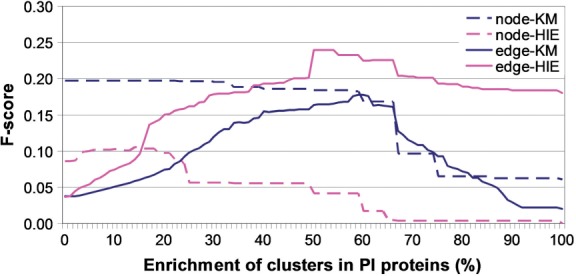
Prediction accuracy for the four clustering methods (node-KM, node-HIE, edge-KM and edge-HIE) in the human PPI network

**Predicting new PI proteins.** When we measure prediction accuracy of the four methods, we define precision as the number of known protein–pathogen associations out of all predicted associations and recall as the number of known associations that we predict out of all known associations. We combine precision and recall into *F*-score. Comparison of the methods with respect to their *F*-scores over the 0-100% enrichment range (see Section 2) is shown in Fig. 4. The larger the enrichment *k*, the higher the prediction confidence. For all *k* above 40%, edge-HIE is the best, followed by node-KM and edge-KM, which are tied, and by node-HIE, which performs poorly. *F*-score trends are different for edge clustering and for node clustering: with edge-HIE and edge-KM, *F*-scores increase up to a certain *k* and then start decreasing; with node-HIE and node-KM, *F*-scores are somewhat ‘uniform’ up to a certain *k* and then suddenly drop.

To assess the statistical significance of these *F*-scores, we compare them with *F*-scores for randomized clusters (see Section 2). In general, the *F*-scores are higher for data clusters than for randomized clusters (Supplementary Fig. S6). The exception is node-HIE: its *F*-scores are higher for randomized clusters for *k* above 60%.

Since each clustering method has its (dis)advantages, the choice of the most appropriate method is application-dependent (Fortunato, 2010). Hence, instead of producing predictions only with the most accurate method (edge-HIE), we produce predictions with each of the methods, except node-HIE, which shows random-like behavior. Since we predict a protein to interact with a pathogen if the enrichment of its cluster is above a given *k*, the choice of *k* is crucial. We decide to use as high *k* as possible while decreasing *F*-score as little as possible. We choose *k* of 66%, since for all three clustering methods, *F*-scores increase or remain relatively ‘uniform’ up to *k* = 66% but then start decreasing. At this *k*, *F*-scores are 23%, 17% and 16% for edge-HIE, node-KM and edge-KM, respectively. These translate into precision of 18% at recall of 30% for edge-HIE, precision of 36% at recall of 11% for node-KM and precision of 16% at recall of 16% for edge-KM. Hence, precision is higher for node-KM than for edge-HIE and edge-KM. However, edge-HIE and edge-KM have higher recall, which might not be surprising, given that they allow for the node overlap between clusters.

Relatively lower *F*-scores should not be alarming for the following reasons. Since the number of known PI proteins will increase in the future, precision, recall and *F*-scores are likely to increase as well. Our predictions are already statistically significant; since we removed clusters with random enrichment in PI proteins, the number of possible predictions is automatically decreased, thus decreasing the *F*-scores. *F*-scores are higher for data clusters than for randomized clusters: for *k* = 66% at which we make predictions, data *F*-scores of 23%, 17% and 16% for edge-HIE, node-KM, and edge-KM are higher than those of 17%, 6% and 12% for their random counterparts, respectively. We demonstrate the superiority of our approach over others (Section 3.1). Finally, our clusters are enriched in biological pathways, unlike their random counterparts, which further validates our approach (Supplementary Section S2).

Supplementary Table S1 lists protein–pathogen associations predicted by edge-HIE, edge-KM and node-KM. Supplementary Fig. S7 shows the overlap of their predictions. Together, they predict 1677 out of 3728 known protein–pathogen associations, resulting in combined recall of 45%. They make the total of 10 190 predictions. Precision is 14% for predictions produced by a single method, 41% for predictions produced by any two methods and 50% for predictions produced by all three methods. Since precision is higher for predictions produced by multiple methods, they could be considered of higher confidence. A small overlap of all three methods (54 predictions) confirms that there is no single ‘best’ method and justifies our decision to use all three methods to produce predictions.

**Literature validation.** We perform literature search for ‘high-scoring’ predictions selected as follows. Of all new predictions, we exclude HIV-related predictions, which would, due to their large number, make literature search difficult. We also exclude predictions involving proteins that could not be found in any article. Of the remaining predictions, we focus on those with cluster enrichment of 100%, resulting in 18 predictions. We validate 44% of the predictions. We link TGFBR2 with vaccinia virus. TGFBR2 transduces TGFB1 signal from the cell surface to the cytoplasm, thus regulating many pathological processes (The UniProt Consortium, 2012) and TGFB1 has explicitly been linked to vaccinia virus (PubMed ID (PMID): 11859112, 16210663). We link BCL-2-like 11 (BCL2L11), BCL-2-like 1 (BCL2L1) and BCL-2 modifying factor (BMF) with vaccinia virus. F1L protein with BCL-2-like structure inhibits apoptosis in vaccinia virus (PMID: 21698224, 18551131). In addition, IL-21 protein, which is related to upregulation of BCL-2 molecules, is critical for response to vaccinia viral infection (PMID: 21257966). We link MCM complex components MCM6, MCM7 and MCM8 with influenza A virus. MCM complex has a role in regulating genome replication of influenza virus (PMID: 17932485). We link NCOA6 with herpes simplex virus. It harbors a potent N-terminal activation domain, which is as active as the herpes simplex virus activation domain (PMID: 10866662).

## 4 CONCLUSION

We introduce edge-GDV-similarity, a sensitive topological measure of edge similarity. When we hierarchically cluster edge-GDV-similar edges in yeast networks, we outperform existing node and edge clustering methods. When we cluster the human network, edge-HIE is better than node-HIE, while edge-KM and node-KM are comparable. Thus, we gain by clustering edges compared to clustering nodes with hierarchical but not *k*-medoids clustering.

We apply our approach to prediction of new PI proteins in human. This is important, since it could suggest candidates for therapeutic intervention. We validate many of our predictions through literature search, which confirms the correctness of our approach.

*Funding:* This work was supported by the National Science Foundation CCF-1243295 grant.

## References

[B1] Ahn Y. (2010). Link communities reveal multiscale complexity in networks. Nature.

[B2] Ashburner M. (2000). Gene Ontology: tool for the unification of biology. Nat. Genet..

[B3] Breitkreutz B.J. (2008). The BioGRID Interaction Database: 2008 update. Nucleic Acids Res..

[B4] Brohee S., van Helden J. (2006). Evaluation of clustering algorithms for protein-protein interaction networks. BMC Bioinformatics.

[B5] Brun C. (2004). Clustering proteins from interaction networks for the prediction of cellular functions. BMC Bioinformatics.

[B6] Chatr-aryamontri A. (2009). VirusMINT: a viral protein interaction database. Nucleic Acids Res..

[B7] Cook S. (1971). The complexity of theorem-proving procedures. Proceedings of the 3rd Annual ACM Symposium on Theory of Computing.

[B8] Dyer M. (2008). The landscape of human proteins interacting with viruses and other pathogens. PLoS Pathog..

[B9] Evans T.S., Lambiotte R. (2009). Line graphs, link partitions, and overlapping communities. Phys. Rev. E Stat. Nonlin. Soft Matter Phys..

[B10] Fortunato S. (2010). Community detection in graphs. Physics Reports.

[B11] Giot L. (2003). A protein interaction map of *Drosophila melanogaster*. Science.

[B12] Goh K.-I. (2007). The human disease network. Proc. Natl Acad. Sci. USA.

[B13] Ho H. (2010). Protein interaction network uncovers melanogenesis regulatory network components within functional genomics datasets. BMC Syst. Biol..

[B14] Jeong H. (2001). Lethality and centrality in protein networks. Nature.

[B15] Jonsson P., Bates P. (2006). Global topological features of cancer proteins in the human interactome. Bioinformatics.

[B16] Kovács I.A. (2010). Community Landscapes: an integrative approach to determine overlapping network module hierarchy, identify key nodes and predict network dynamics. PLoS One.

[B17] Kuchaiev O. (2010). Topological network alignment uncovers biological function and phylogeny. J. R. Soc. Interface.

[B18] LaCount D. (2005). A protein interaction network of the malaria parasite plasmodium falciparum. Nature.

[B19] Memisević V. (2010). Complementarity of network and sequence information in homologous proteins. J. Integr. Bioinform..

[B20] Milenković T. (2010). Systems-level cancer gene identification from protein interaction network topology applied to melanogenesis-related interaction networks. J. R. Soc. Interface.

[B21] Milenković T. (2011). Dominating biological networks. PLoS One.

[B22] Milenković T. (2010). Optimal network alignment with graphlet degree vectors. Cancer Inform..

[B23] Milenković T., Pržulj N. (2008). Uncovering biological network function via graphlet degree signatures. Cancer Inform..

[B24] Newman M.E.J. (2004). Fast algorithm for detecting community structure in networks. Phys. Rev. E.

[B25] Palla G. (2005). Uncovering the overlapping community structure of complex networks in nature and society. Nature.

[B26] Parrish J. (2007). A proteome-wide protein interaction map for Campylobacter jejuni. Genome Biol..

[B27] Peri S. (2004). Human protein reference database as a discovery resource for proteomics. Nucleic Acids Res..

[B28] Pržulj N. (2007). Biological network comparison using graphlet degree distribution. Bioinformatics.

[B29] Radivojac P. (2008). An integrated approach to inferring gene-disease associations in humans. Proteins.

[B30] Rosvall M., Bergstrom C.T. (2008). Maps of random walks on complex networks reveal community structure. Proc. Natl Acad. Sci. USA.

[B31] Sharan R., Ideker T. (2008). Protein networks in disease. Genome Res..

[B32] Sharan R. (2007). Network-based prediction of protein function. Mol. Syst. Biol..

[B33] Simonis N. (2009). Empirically controlled mapping of the Caenorhabditis elegans protein-protein interactome network. Nat. Methods.

[B34] Stelzl U. (2005). A human protein-protein interaction network: a resource for annotating the proteome. Cell.

[B35] The UniProt Consortium (2012). Reorganizing the protein space at the Universal Protein Resource (UniProt). Nucleic Acids Res..

[B36] Vanunu O. (2010). Associating genes and protein complexes with disease via network propagation. PLoS Comput. Biol..

[B37] Watts D., Strogatz S. (1998). Collective dynamics of ‘small-world’ networks. Nature.

[B38] Yu H. (2008). High-quality binary protein interaction map of the yeast interactome network. Science.

